# AtrR Is an Essential Determinant of Azole Resistance in Aspergillus fumigatus

**DOI:** 10.1128/mBio.02563-18

**Published:** 2019-03-12

**Authors:** Sanjoy Paul, Mark Stamnes, Grace Heredge Thomas, Hong Liu, Daisuke Hagiwara, Katsuya Gomi, Scott G. Filler, W. Scott Moye-Rowley

**Affiliations:** a Department of Molecular Physiology and Biophysics, Carver College of Medicine, University of Iowa, Iowa City, Iowa, USA; b Division of Infectious Diseases, Los Angeles Biomedical Research Institute at Harbor-UCLA Medical Center, Torrance, California, USA; c David Geffen School of Medicine at UCLA, Los Angeles, California, USA; d Faculty of Life and Environmental Sciences, University of Tsukuba, Ibaraki, Japan; e Graduate School of Agricultural Science, Tohoku University, Sendai, Japan; Universidade de São Paulo

**Keywords:** ABC transporters, antifungal resistance, *Aspergillus fumigatus*, molecular genetics, transcriptional regulation

## Abstract

Aspergillosis associated with azole-resistant Aspergillus fumigatus has a mortality rate that can approach 90% in certain patient populations. The best-understood avenue for azole resistance involves changes in the *cyp51A* gene that encodes the target of azole drugs, lanosterol α-14 demethylase. The most common azole resistance allele currently described is a linked change corresponding to a change in the coding sequence of *cyp51A* and a duplication of a 34-bp region in the promoter leading to a tandem repeat (TR). Our previous studies identified a positively acting transcription factor called AtrR that binds to the promoter of *cyp51A* as well as that of an important membrane transporter protein gene called *abcG1*. In this work, we characterize two different mutant alleles of *atrR*, either an overproducing or an epitope-tagged form, causing constitutive activation of this factor. Using an epitope-tagged allele of *atrR* for chromatin immunoprecipitation coupled with high-throughput sequencing (ChIP-seq), the genomic binding sites for AtrR were determined. Close to 900 genes were found to have an AtrR response element (ATRE) in their promoter regions. Transcriptome evaluation by RNA sequencing (RNA-seq) indicated that both alleles led to elevated transcription of a subset of target genes. An electrophoretic mobility shift assay and DNase I protection mapping localized the ATREs in both the *abcG1* and *cyp51A* promoters. The ATRE in *cyp51A* was located within the 34-bp repeat element. Virulence in a murine model was compromised when AtrR was either deleted or overproduced, indicating that the proper dosage of this factor is key for pathogenesis.

## Abstract (precis)

Aspergillus fumigatus is the major filamentous fungal pathogen in humans. Infections associated with A. fumigatus are often treated with azole drugs, but resistance to these antifungal agents is increasing. Mortality from aspergillosis associated with azole-resistant fungi is extremely high. Previous work has identified transcriptional control of the azole drug target-encoding gene *cyp51A* as an important contributor to resistance in A. fumigatus. Here, we demonstrate that the transcription factor AtrR binds to a region in the *cyp51A* promoter that is associated with alleles of this gene conferring clinically important azole resistance. Using high-throughput genomic technologies, we also uncover a large suite of target genes controlled by AtrR. These data indicate that AtrR coordinately regulates many different processes involved in drug resistance, metabolism, and virulence. Our new understanding of AtrR function provides important new insight into the pathogenesis of A. fumigatus.

## INTRODUCTION

Infections associated with azole-resistant forms of Aspergillus fumigatus have a mortality rate that can approach 90% in particular patient populations (discussed in reference 1). This places a high priority on understanding the molecular basis of development of this problematic phenotype. Extensive studies from several laboratories have provided support for the prevalence of a compound mutation in the *cyp51A* gene that encodes the target enzyme of azole drugs, lanosterol α-14 demethylase (recently reviewed in reference 2). This interesting compound mutation consists of a change within the *cyp51A* open reading frame that replaces a leucine at position 98 with a histidine residue (L98H) and contains a duplication of a 34-bp region in the promoter (tandem repeat of 34 bp [TR_34_]) (3). These linked changes cause elevated *cyp51A* mRNA levels and produce an altered enzyme that is believed to have a defect in azole drug binding (4, 5). Both of these alterations must be present for the synergistic increase in azole resistance to occur (6). Other alterations in the *cyp51A* promoter that duplicate similar regions have been described, with these changes sharing the common feature of elevating transcription of *cyp51A* (7, 8).

The requirement for increased transcription of *cyp51A* makes analyzing the transcriptional control mechanisms of this gene an important step in developing strategies to interfere with acquisition of azole resistance. A recent detailed analysis has provided new insights into the function of both the wild-type *cyp51A* promoter and the TR_34_ derivative (9). A binding site for the important positive transcriptional regulator of ergosterol biosynthetic enzyme-encoding genes called SrbA was found to bind to the 34-bp region, and this binding element was designated the SrbA response element (SRE) (9). Two SREs were localized to the 34-bp region and shown to be duplicated in the TR_34_ mutation. Additional regulatory sites have been documented for the negatively acting factors CCAAT-binding complex (CBC) and HapX (10). The CBC is a complex of three proteins (HapB, HapC, and HapE) (reviewed in reference 11) that act along with HapX to repress the transcription of *cyp51A* and a number of other loci encoding enzymes required for ergosterol production and iron homeostasis (12). Mutation of either the *hapE* or *hapX* gene led to elevated azole resistance and increased *cyp51A* transcription (9).

A new participant in the transcriptional control of *cyp51A* gene expression was recently discovered to be a Zn_2_Cys_6_ cluster-containing transcription factor that was able to elevate azole resistance when overproduced in Aspergillus oryzae (13). This factor, designated ABC transporter regulator, or AtrR, was found to be present in A. fumigatus and to be required for normal azole tolerance. AtrR was linked to regulation of both *cyp51A* and the ATP-binding cassette (ABC) transporter-encoding gene *abcG1* (also known as [aka] *cdr1B*) by two pieces of evidence. First, strains lacking *atrR* failed to drive normal transcription of either *cyp51A* or *abcG1*. Second, chromatin immunoprecipitation (ChIP) experiments demonstrated that AtrR bound to both the *cyp51A* and *abcG1* promoter regions. However, the sequence of the AtrR response element (ATRE), its relationship to the SRE, and the genomic target sites of this factor remained among the gaps in our knowledge.

Here, we report the generation of two hypermorphic or hyperactive alleles of *atrR,* as well as identification of the suite of AtrR-responsive genes *in vivo* using chromatin immunoprecipitation coupled with high-throughput DNA sequencing (ChIP-seq). The sequence of the ATRE was identified from these analyses and confirmed by a combination of DNase I footprinting, electrophoretic mobility shift assays (EMSA), and mutagenesis. AtrR and SrbA both bind to the critical 34-bp region of *cyp51A* and are likely to cooperate to drive the transcription of this key azole resistance determinant, while only AtrR appears to control *abcG1* transcription.

## RESULTS

### Generation of two hypermorphic alleles of *atrR*.

**FIG 1:**
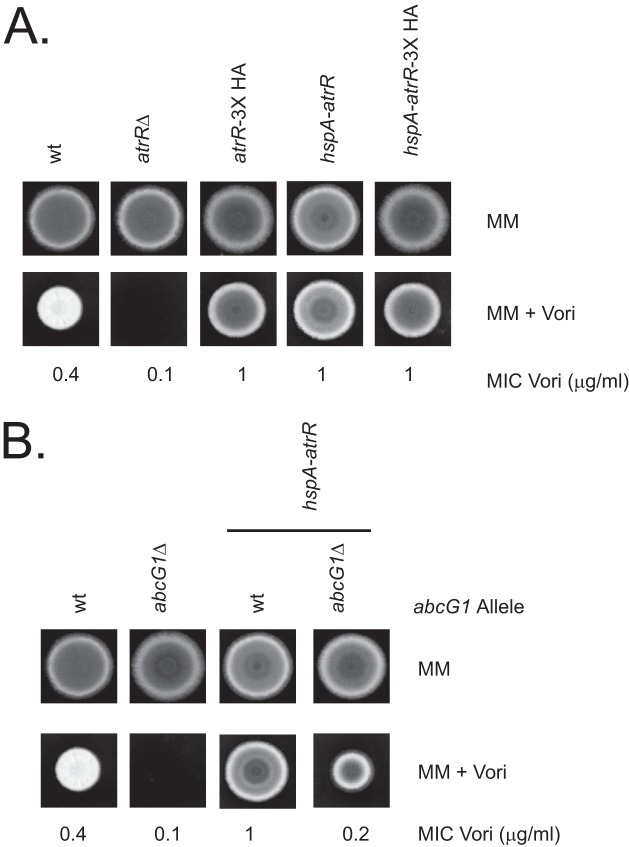
Azole resistance phenotypes of *atrR* alleles. (A) Isogenic wild-type (wt) AfS35 strains were produced that contained the indicated forms of the *atrR* gene. The wild-type *atrR* gene was replaced with the hygromycin resistance gene (*atrR*Δ) or a 3× HA epitope tag inserted at its C terminus (*atrR*-3× HA). The strong *hspA* promoter region was used to drive either the wild-type *atrR* coding sequence (*hspA*-atrR) or the epitope-tagged allele (*hspA-atrR*-3× HA). Equivalent numbers of spores from representative transformants were prepared and spotted on solid minimal medium (MM) or the same medium containing voriconazole (MM + Vori). Plates were incubated at 37°C and then photographed. Corresponding MICs for voriconazole (MIC Vori) were determined for each strain and are listed below in micrograms per milliliter. (B) Spore suspensions were prepared from wild-type and *abcG1*Δ cells containing an *hspA-atrR* fusion gene where indicated or retaining the wild-type *atrR* locus. Voriconazole resistance was assayed as described above.

We previously generated an epitope-tagged form of *atrR* corresponding to the wild-type gene containing a C-terminal insertion of a 3× hemagglutinin (3× HA) tag (13). More recently, we replaced the endogenous *atrR* promoter with the cognate region from the highly transcribed *hspA* gene (14). Based on RNA-seq analyses (15), we estimate that this *hspA* allele has the potential to overproduce *atrR* mRNA by at least 10-fold. To compare the functions of these different forms of *atrR*, we placed appropriate transformants on minimal medium lacking or containing 0.1 μg/ml voriconazole. We also included isogenic wild-type *atrR*Δ null cells for comparison along with an *hspA-atrR*-3× HA allele used earlier. Plates were incubated at 37°C to evaluate the relative resistance conferred by these different *atrR* alleles (Fig. 1A).

The presence of the *hspA* promoter upstream of *atrR*, either tagged with 3× HA or untagged, strongly elevated voriconazole resistance. Interestingly, even insertion of the 3× HA epitope tag alone was sufficient to elevate azole resistance. These data suggest that fusion of foreign sequences to the C terminus or overproduction of AtrR (see below) both represent means of overcoming the normal control of transactivation by this transcription factor.

Work from several labs has implicated an ATP-binding cassette (ABC) transporter-encoding gene, here called *abcG1*, as being an important determinant in azole resistance (16, 17), as well as being a target gene for AtrR regulation (13). To examine the epistatic relationship between AtrR and *abcG1*, we constructed isogenic *hspA-atrR* strains that contained or lacked a copy of *abcG1*. We compared these strains for resistance to voriconazole as described above along with wild-type and *abcG1*Δ control strains (Fig. 1B).

A loss of *abcG1* strongly depressed voriconazole resistance in a strain overproducing AtrR. However, even in the absence of *abcG1*, overproduction of AtrR was still able to elevate azole resistance. This is consistent with *abcG1* being an important but not exclusive target gene of AtrR impacting voriconazole resistance.

### Expression of *abcG1* responds to hypermorphic alleles of *atrR*.

To confirm that increased AtrR levels led to increased expression of AbcG1, we directly assayed the expression of these two proteins using polyclonal antibodies directed against recombinant forms of each. Isogenic wild-type and *atrR*Δ strains were grown in minimal medium, along with transformants containing the epitope-tagged *atrR* allele (*atrR*-3× HA) or strains with the *hspA* promoter in place of the wild-type *atrR* promoter driving the native (*hspA-atrR*) or epitope-tagged (*hspA-atrR*-3× HA) allele. Whole-cell protein extracts were prepared from these strains and analyzed by Western blotting using anti-AtrR or anti-AbcG1 antiserum.

**FIG 2:**
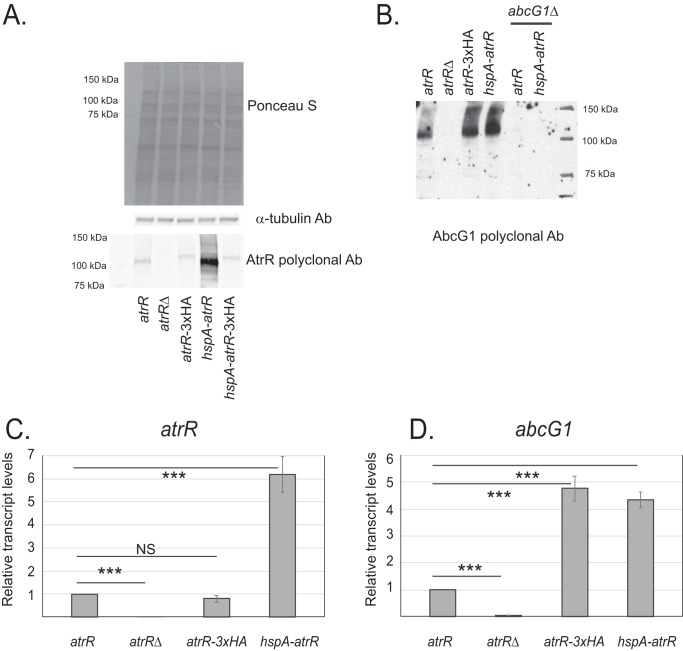
Regulation of expression by AtrR. (A) A rabbit polyclonal directed against the AtrR DNA-binding domain was generated against a recombinant form of this protein domain. Whole-cell protein extracts were prepared from isogenic wild-type (*atrR*), *atrR*Δ mutant, *atrR*-3× HA mutant, *hspA-atrR* mutant, or *hspA-atrR*-3× HA mutant strains. Equal amounts of protein were resolved on SDS-PAGE gel and then transferred to nitrocellulose membranes. These membranes were stained with Ponceau S to ensure equal loading and then analyzed by Western blotting using an anti-tubulin antibody (Ab) and the anti-AtrR antibody. Molecular mass standards are indicated on the left side of the panel. (B) Whole-cell protein extracts were prepared from the indicated strains and analyzed by Western blotting using anti-AbcG1 antiserum (16). (C) RNA levels for *atrR* were assessed using qRT-PCR. Total RNA was prepared from the strains listed at the bottom and *atrR* mRNA measured using specific primers. Actin mRNA was used as an internal standard, and RNA levels are normalized those produced in wild-type cells. *P* values were determined by an unpaired 2 tailed *t*-test and are reported above each comparison as NS, not significant; *, *P* < 0.05; **, *P* < 0.005; ***, *P* < 0.001. (D) Levels of *abcG1* mRNA were determined via qRT-PCR.

AtrR was seen as a 100-kDa protein in extracts from wild-type cells that was eliminated in *atrR*Δ cells (Fig. 2A). The presence of the *hspA* promoter in place of the wild-type *atrR* promoter led to the overproduction of AtrR. The epitope-tagged form of AtrR was expressed at levels similar to those in the wild type, irrespective of being under wild-type or *hspA* promoter control. Our use of the anti-AtrR antibody allowed facile comparison of levels of tagged and untagged transcription factors. The size of this polypeptide was increased by the presence of the 3× HA tag, as expected; however, the steady-state level of this form of AtrR was not increased when under *hspA* promoter control, unlike the effect seen for the wild-type factor. These data confirm that the wild-type protein was overproduced when driven from the *hspA* promoter, as suggested above.

The expression of AbcG1 was strongly responsive to the presence of *atrR* (Fig. 2B), consistent with earlier data (13). Both overproduction of the native AtrR protein and the presence of the epitope-tagged *atrR* allele increased the expression of AbcG1.

We next measured expression levels of the mRNA for *atrR* and *abcG1* to determine if these changes at the protein level correlated with the expected alterations in gene transcription. Wild-type and *atrR*Δ, *atrR*-3× HA, and *hspA-atrR* mutant strains were grown to mid-log phase, total RNA was prepared, and quantitative reverse transcription-PCR (qRT-PCR) was used to evaluate mRNA levels for *atrR* and *abcG1*.

The transcription of *atrR* was increased in the presence of the *hspA-atrR* allele but was not altered by the insertion of the epitope tag at the C terminus of the native gene (Fig. 2C). In contrast, *abcG1* transcription was similarly increased by the presence of either hypermorphic allele of *atrR* (Fig. 2D). We suggest from these data that the causes of the increased *abcG1* expression induced by the presence of the epitope-tagged or the *hspA*-driven allele of *atrR* are different and discuss this more fully below.

### Genomic target genes of AtrR.

**FIG 3:**
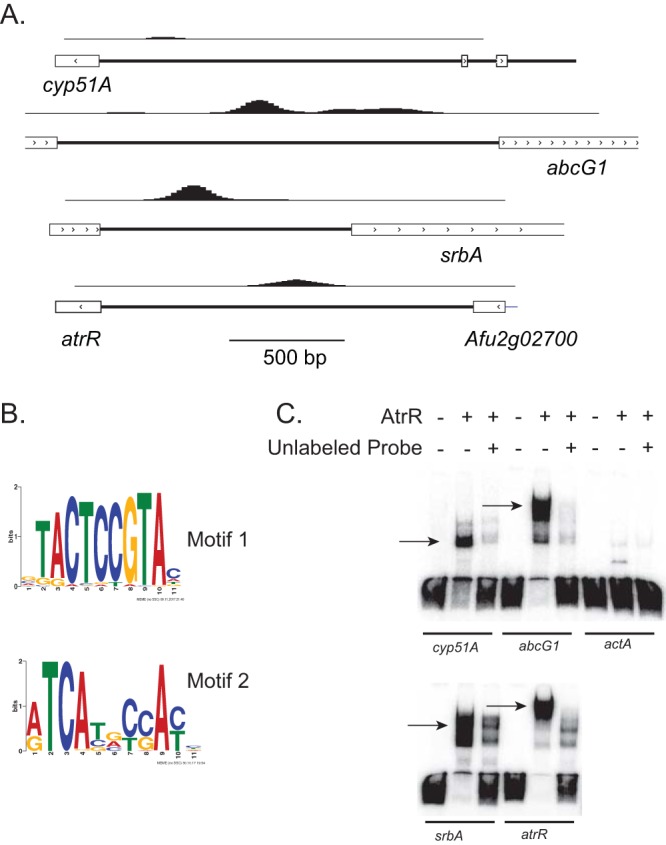
DNA site selection by AtrR. (A) Chromatin immunoprecipitation–high-throughput sequencing (ChIP-seq) was used to profile genomic sites of AtrR-3× HA binding. ChIP-seq profiles of several selected genes are shown as diagrams of the read density in their respective promoter regions (top line of each pair) along with a map of the genes around these elements. The direction of gene transcription is indicated by the arrows within the boxed exons. The scale of these diagrams is indicated at the bottom by the line labeled 500 bp. (B) MEME-ChIP analysis of binding sites enriched in ChIP-seq peaks is shown as a MEME logo. Higher levels of conservation are indicated by the larger letters. Motif 1 is likely to be an AtrR response element (ATRE), while motif 2 is an SrbA response element (SRE). (C) Electrophoretic mobility shift assays (EMSA) of regions containing putative ATREs located upstream of the indicated genes. Probes from each gene were prepared by PCR and end-labeled with [γ-^32^P]-ATP and T4 polynucleotide kinase. Purified probe fragments were mixed with recombinant AtrR in the presence or absence of a molar excess of the unlabeled probe. After binding, these reaction mixtures were separated by nondenaturing 5% acrylamide PAGE gels, the gels were dried, and labeled fragments were detected by use of a Typhoon imager. AtrR-DNA complexes are indicated by the arrows.

We have previously shown that AtrR binds directly to the promoters of *abcG1* and *cyp51A in vivo* by single-gene chromatin immunoprecipitation (ChIP) analyses (13). To determine the range of target genes of AtrR in the entire A. fumigatus genome, we carried out ChIP coupled with high-throughput sequencing (ChIP-seq) using the *atrR*-3× HA and *hspA-atrR*-3× HA strains. Wild-type (negative control), *atrR*-3× HA, and *hspA-atrR*-3× HA strains were grown to mid-log phase and cross-linked chromatin prepared, as described previously (18). Isolated chromatin prepared for next-generation sequencing using standard methods (19) and the sequencing reads were mapped to the A. fumigatus genome sequence. Significantly increased read depth peaks were called by comparison to untagged wild-type controls and assessed for proximity to annotated protein-coding genes. We found nearly 900 peaks for AtrR binding in the promoter regions of protein-coding genes. Plots of representative ChIP densities are shown in Fig. 3A.

As expected from our single-gene ChIP data (13), we found significant AtrR binding to regions upstream of both *abcG1* and *cyp51A*. Interestingly, we also found evidence for AtrR binding to the promoter regions of both the *srbA* and *atrR* genes themselves. The *srbA* gene encodes the previously characterized sterol response transcription factor SrbA (20), while AtrR binding to its own promoter suggests the possibility that *atrR* is autoregulated.

As the specific element recognized by AtrR is not well understood, we compared the sequences of all the detected ChIP-seq peaks using the algorithm MEME-ChIP (21). This algorithm compares sequences surrounding each of the annotated peak summits to search for a shared common element. This analysis predicted the presence of two most common shared elements shown in Fig. 3B. We believe that motif 1 represents the half-site of an AtrR response element (ATRE), while motif 2 corresponds to the binding site for the SrbA transcription factor (SrbA response element [SRE]). Motif 1 was present in approximately 600 of the ChIP-seq peaks, while motif 2 was found in ∼400 peaks. Interestingly, these were present at the same promoter in roughly 100 genes. These sequences are analyzed in more detail below.

We next validated that DNA regions containing these different ATREs could be bound by a recombinant form of AtrR corresponding to the N-terminal DNA-binding domain of the factor. In all cases, each probe was produced by PCR amplification from A. fumigatus genomic DNA, radiolabeled, and incubated with the bacterially produced AtrR alone or in the presence of a 40-fold molar excess of the unlabeled probe. We also used a region from the promoter of the actin-encoding *actA* gene as a control for nonspecific binding. After formation of protein-DNA complexes, these reaction mixtures were separated on a nondenaturing 5% polyacrylamide gel and detected by autoradiography (Fig. 3C).

AtrR was able to bind to all these target genes containing ATREs, while no binding was seen to the *actA* promoter. These data support the coincidence of ChIP-seq binding peaks and bona fide ATREs in the A. fumigatus genome.

Comparison of the functions of the proteins produced from these potential regulatory targets of AtrR indicated that several gene ontology (GO) terms were enriched. The most enriched GO terms included sequence-specific DNA binding, plasma membrane, and several metabolic processes (see Fig. S1 in the supplemental material). The list of sequence-specific DNA-binding protein-encoding genes consisted of 42 genes and included loci previously shown to influence azole resistance, such as *srbA* (22), *srbB* (18), and *yap1* (23). This large number (42) (see Table S1) of transcriptional regulators suggests the possibility that their regulation by AtrR might lead to an amplification of the effect of AtrR through the action of these downstream regulators, a suggestion we support below.

10.1128/mBio.02563-18.1 FIG S1 GO term enrichment for AtrR target genes. Output from FungiFun2 (Priebe et al. [42], #2727) of genes that contain an AtrR response element (ATRE) by ChIP-seq analysis. Numbers refer to the number of genes in each category. Download FIG S1, PDF file, 0.8 MB. Copyright © 2019 Paul et al. 2019 Paul et al. This content is distributed under the terms of the Creative Commons Attribution 4.0 International license.

10.1128/mBio.02563-18.3 TABLE S1 Sequence-specific transcriptional regulation bound by AtrR in *Aspergillus fumigatus*. This table represents all known transcription factor-encoding genes that were found to have AtrR binding in their promoter regions via the ChIP-seq experiments reported here. The log_2_ expression values are from our RNA-seq data and refer to the ratio of the RNA levels from *atrR*-3× HA (3× HA)/wt, *hspA-atrR* (*hspA*)/wt, or *atrR*Δ (*atrR* null)/wt. Download Table S1, XLSX file, 0.1 MB. Copyright © 2019 Paul et al. 2019 Paul et al. This content is distributed under the terms of the Creative Commons Attribution 4.0 International license.

**FIG 4:**
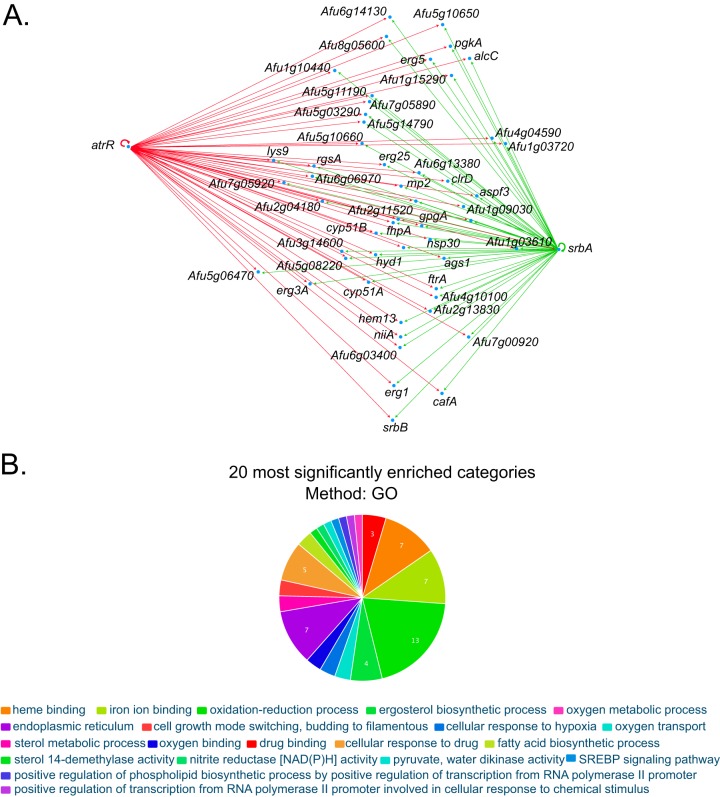
Shared target genes of AtrR and SrbA. (A) A diagram representing the genes that contain both an ATRE as predicted from the ChIP-seq data reported here and a SRE as found in reference 18. (B) FungiFun2 (42) was used to identify GO terms enriched in these genes. SREBP, SRE binding protein.

Previous ChIP-seq experiments have identified the potential targets for SrbA gene regulation (18). Since *cyp51A* has already been demonstrated to be a target gene for both SrbA and AtrR regulation (13), we wondered if other genes might be regulated by both AtrR and SrbA (Fig. S2). Comparing the ChIP-seq data for these factors, we found that 51 genes were present in lists of ChIP-seq-positive targets for both AtrR and SrbA (Fig. 4A). These include ergosterol biosynthetic genes like *cyp51A*, *cyp51B*, *erg3A*, *erg5,* and *erg25* (recently reviewed in reference 2), transcription factors such as *srbA* itself and *srbB*, iron uptake (*ftrA*) (24), and a heme biosynthesis gene (*hem13*) (20). As the total number of SrbA-regulated genes was estimated to be 126, almost 50% of these are also predicted to contain ATREs, indicating extensive overlap between these two transcription factors. This extensive overlap helps explain why the SRE was the second most common enriched motif found in our MEME-ChIP analysis.

10.1128/mBio.02563-18.2 FIG S2 GO term enrichment for SrbA unique targets. Output of FungiFun2 of genes found in SrbA ChIP-seq analysis that are only predicted to contain a SrbA response element (SRE). Data for SrbA are from (Chung et al. [18], #2590). Download FIG S2, PDF file, 0.8 MB. Copyright © 2019 Paul et al. 2019 Paul et al. This content is distributed under the terms of the Creative Commons Attribution 4.0 International license.

### Transcriptomic profiling of hyperactive alleles of *atrR*.

**FIG 5:**
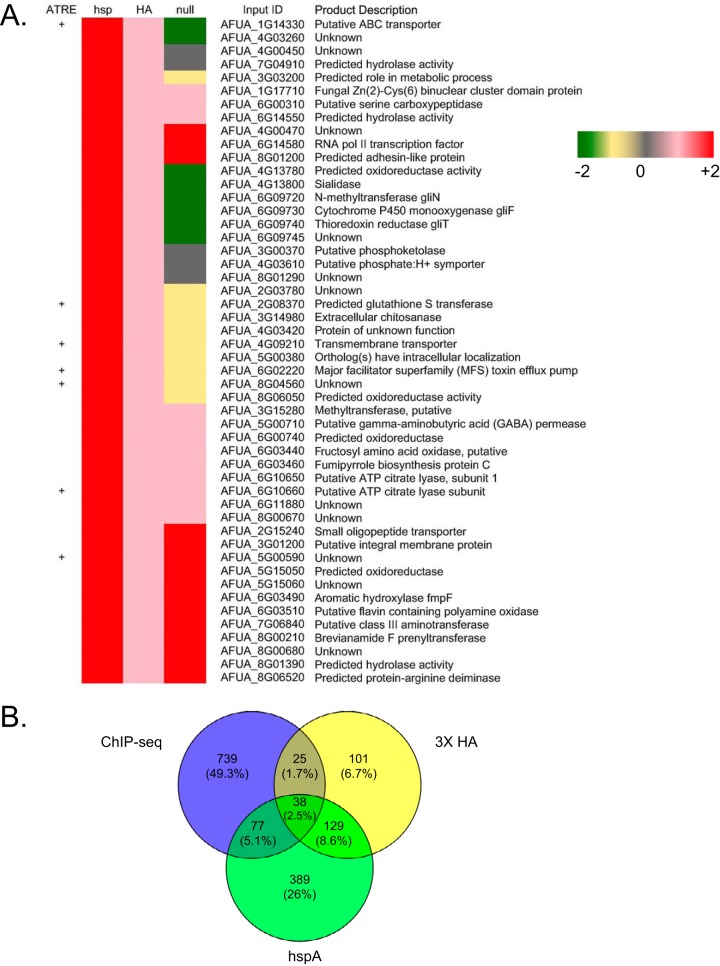
RNA-seq analysis of *atrR* alleles. (A) A heat map of the RNA-seq data obtained from use of RNA prepared from isogenic wild-type, *atrR*Δ (null) mutant, *hspA-atrR* (*hspA*) mutant, and *atrR*-3× HA (HA) strains is shown. Colors represent the log_2_ values of the ratio of RNA levels detected in the indicated strains at the top relative to those present in the wild-type cells. The presence of an ATRE in the promoter region of each gene is indicated by the + symbol. (B) Venn diagram showing the union of all ChIP-seq-positive genes with the genes corresponding to transcripts overproduced by a log_2_ of 1 or greater in strains containing either hyperactive allele of *atrR*. The percent value represents the portion each category makes up of the total number of genes analyzed here. pol, polymerase.

Since a large number of genes were predicted to have binding sites for AtrR in their promoters, we wanted to determine the range of genes that responded to the two different hyperactive alleles, the *hspA* overproduction derivative and the 3× HA-tagged form. Isogenic wild-type and *atrR*Δ, *hspA-atrR,* and *atrR*-3× HA mutant strains were grown in minimal medium. Total RNA was prepared and analyzed by RNA-seq, as described previously (15). The top 50 upregulated genes clustered by regulatory pattern are shown in Fig. 5A.

A total of 633 genes were upregulated by at least 2-fold in the presence of the *hspA-atrR,* while the epitope-tagged allele exhibited 293 genes that were upregulated by this same degree. Importantly, only around 20% of these upregulated genes were also found to be positive for the presence of an ATRE as assessed by ChIP-seq. The most highly induced gene in the presence of either hyperactive allele was the *abcG1* gene. GO terms for the top 100 genes in the cluster analysis showed enrichment for genes involved in oleate metabolism and gliotoxin biosynthesis, but these two groups only involved 5 genes total (data not shown). We also compared the effects seen when *atrR* was deleted with results from our earlier study (13). Of the 200 genes that showed the greatest reduction in expression upon loss of *atrR*, only approximately 10% were seen to overlap between the current RNA-seq measurement and in the previous assay (13). This is likely due to cells being grown as a biofilm in these experiments, while in the previous assays, cells were grown planktonically. Large differences in transcription have previously been reported in a comparison of genes grown under these different conditions (15).

The overlap between upregulated genes in either hyperactive allele was compared along with the total number of ChIP-positive genes (Fig. 5B). Only 38 (2.5% of the total) genes were upregulated in the presence of one or both hyperactive alleles and contained ATREs. Additionally, the effect of overproduction of wild-type AtrR (in the *hspA-atrR* fusion) had a stronger phenotypic response than did the presence of the *atrR*-3× HA allele, as evidenced by the larger number of genes that were induced in response to the *hspA-atrR* allele. These data support the view that much of the AtrR-responsive regulon occurs via indirect effects through the engagement of other transcription factor networks (like SrbA) and others identified in the ChIP-seq analysis described above. This also supports the suggestion made above that the effects of AtrR are amplified via induction of the large number of transcription factor-encoding genes containing ATREs.

### A hyperactive allele of *atrR* cannot suppress phenotypes of a *srbA*Δ strain.

**FIG 6:**
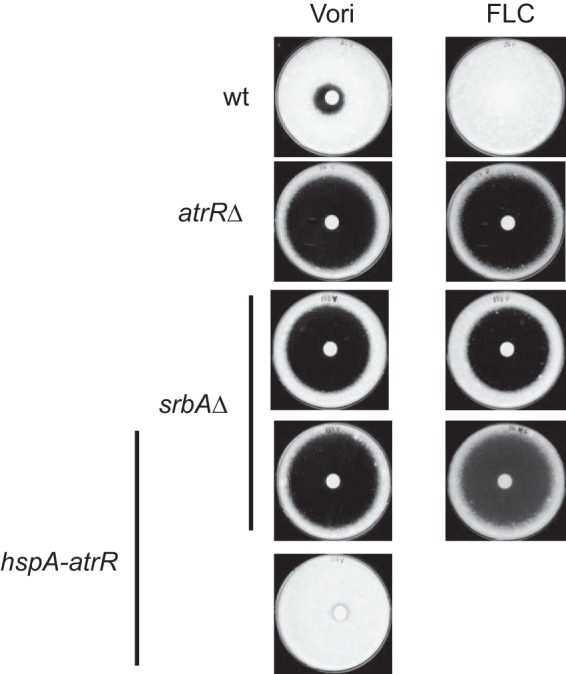
Hypermorphic *atrR* allele cannot suppress the phenotype of a *srbA*Δ strain. The indicated strains were analyzed using a zone of inhibition test employing the azole drugs listed (Vori, voriconazole; FLC, fluconazole).

While the data described above provided several links between AtrR and SrbA functions, we wanted to examine the interaction of these two important genes that impact related *in vivo* processes. We employed genetic epistasis to determine if the hypermorphic *hspA*-*atrR* allele was capable of suppressing the well-described azole sensitivity of a *srbA*Δ null strain. Isogenic wild-type and *atrR*Δ, *srbA*Δ, *hspA-atrR,* and *hspA-atrR srbA*Δ mutant strains were grown and tested using a disk diffusion assay. Plates were incubated at 37°C and photographed (Fig. 6).

While overproduction of AtrR strongly elevated voriconazole resistance in an otherwise wild-type genetic background, the loss of *srbA* completely blocked this effect. The epistasis of *srbA* to the *hspA-atrR* allele was also seen when testing for fluconazole resistance or hypoxic growth (data not shown). Both of these genes are required for normal azole resistance and driving high-level expression of AtrR cannot suppress the need for SrbA under these conditions.

### Interaction of AtrR with the *cyp51A* 34-bp element.

**FIG 7:**
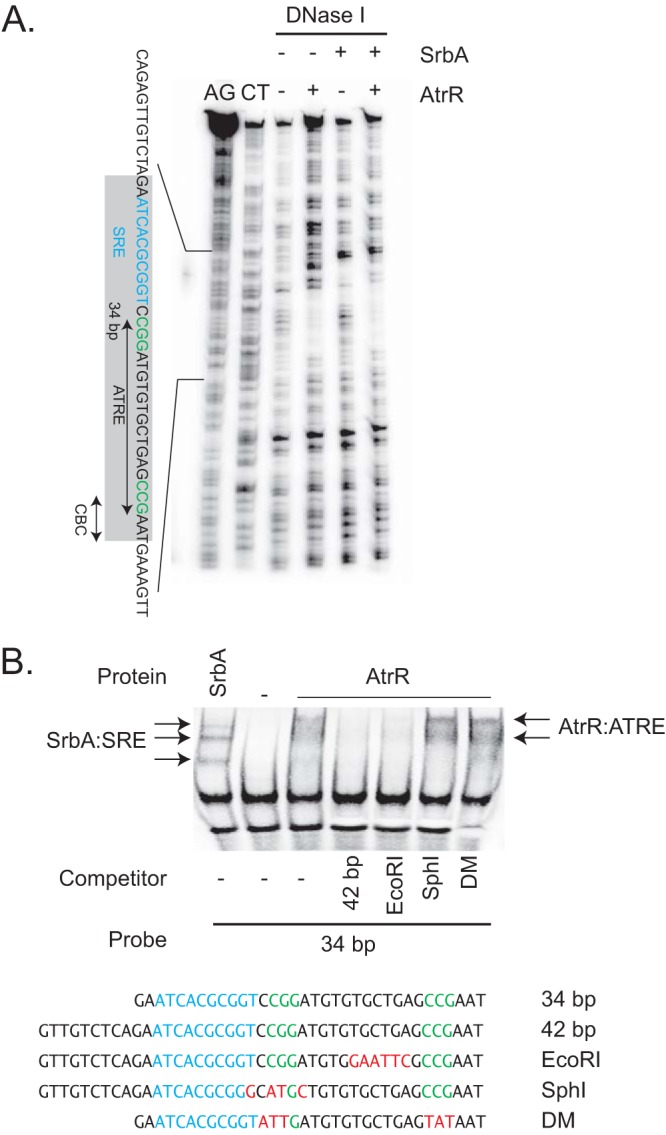
AtrR binding to *cyp51A*. (A) A 5′-end-labeled probe corresponding to the *cyp51A* promoter region was prepared and used in Maxam-Gilbert chemical sequencing reactions (AG, purine residues; CT, pyrimidine residues) to generate a size standard to allow localization of the binding sites for AtrR and SrbA. This same probe was then used in a standard DNase I protection assay with the addition of recombinant forms of AtrR or SrbA where indicated. The drawing on the left represents the sequence elements of interest. The gray bar denotes the bounds of the 34-bp region that is duplicated in the TR_34_ promoter mutation. The double-headed arrow labeled CBC indicates the previously shown binding site for the CAAT-binding complex (9), and the sequence in blue represents a previously mapped SrbA response element (SRE) from this same earlier study. The AtrR response element (ATRE) is indicated with the two CGG repeats in green text. (B) Electrophoretic mobility shift assay (EMSA) analysis of sequence requirements for AtrR binding. A biotin-labeled form of the 34-bp element from *cyp51A* was used as a probe for all these EMSA reactions. Recombinant forms of SrbA or AtrR were added to this probe in the absence or presence of the indicated nonbiotinylated competitor DNAs listed at the bottom. Protein-DNA complexes formed by SrbA. The locations of the SRE and ATRE are shown as in panel A, with mutant residues indicated by red text. All competitors were present at 10-fold molar excess.

While the ChIP-seq data implicated a region in the 34-bp region of the *cyp51A* promoter as the binding site for AtrR, we wanted to precisely locate this element and support our identification of the ATRE. Additionally, we wanted to compare where SrbA bound to the 34-bp repeat with the ATRE and previous data using a plasmon resonance approach (9). We used our recombinant forms of SrbA and AtrR in a DNase I protection assay on the *cyp51A* promoter region. Purine- and pyrimidine-specific reactions were also carried out on this same probe to provide a means of mapping protected areas back to the DNA sequence. Limited DNase I digestions were performed on the free probe or this same probe bound to SrbA and/or AtrR. All samples were then electrophoresed through denaturing acrylamide gels and DNA fragments detected by autoradiography (Fig. 7A).

AtrR protected a roughly 25-bp region containing the ATRE from DNase I cleavage. This ATRE was located immediately upstream of the CBC-binding site mapped earlier (9). SrbA bound to an SRE that was previously mapped and designated SRE1. A downstream SRE that overlapped with the ATRE, called SRE2, was not protected from DNase I cleavage in this assay. This likely reflects the finding that the *K_d_* (dissociation constant) for SrbA binding to SRE2 is 10-fold higher than for SRE1 (9). Together, these data suggest that only SRE1 may play a relevant role *in vivo*.

To probe the specific sequence elements in the ATRE that are important for AtrR recognition, we used an electrophoretic mobility shift assay (EMSA) with the 34-bp region of *cyp51A* as the probe. This was chemically synthesized with a biotin moiety attached to the 5′ end of each oligonucleotide used to generate the probe. We also produced nonbiotinylated competitor oligonucleotides corresponding to a mutant with a 6-bp substitution in the region between the two CCG repeats (EcoRI), a 4-bp replacement mutant in the upstream CCG repeat (SphI), or with a mutant lacking both copies of the CCG repeat (DM). These unlabeled oligonucleotides were used as competitors to determine if they retained the ability to block AtrR binding to the biotinylated 34-bp probe. Recombinant proteins were added to the biotinylated probe in the absence or the presence of the indicated competitor DNAs. After binding, free probe and protein-probe complexes were resolved on nondenaturing acrylamide gels, transferred to nylon membranes, and visualized by blotting with an IRDye-labeled streptavidin (Fig. 7B).

AtrR formed a complex with the 34-bp probe that could be competed away using either a 42-bp version of this promoter fragment or the same 42-bp competitor containing the 6-bp mutation between the two CCG repeats. Importantly, mutant competitors lacking either one (SphI) or both CCG repeats (DM) could not compete. These data confirm the importance of these CCG repeats in normal DNA binding of the ATRE by AtrR.

### A region containing tandem ATREs is important for normal *abcG1* expression.

We previously described the use of a firefly luciferase reporter system to compare expression of the *abcG1* gene and a homologue called *abcG4* (Afu2g15130). The region 1,000 bp upstream of the ATG codon for both these genes was used to drive luciferase expression, and the resulting fusion constructs produced similar low-level luciferase expression (14). This was surprising since qRT-PCR analyses of these same gene indicated that *abcG1* mRNA was 4-fold higher than that of *abcG4*.

**FIG 8:**
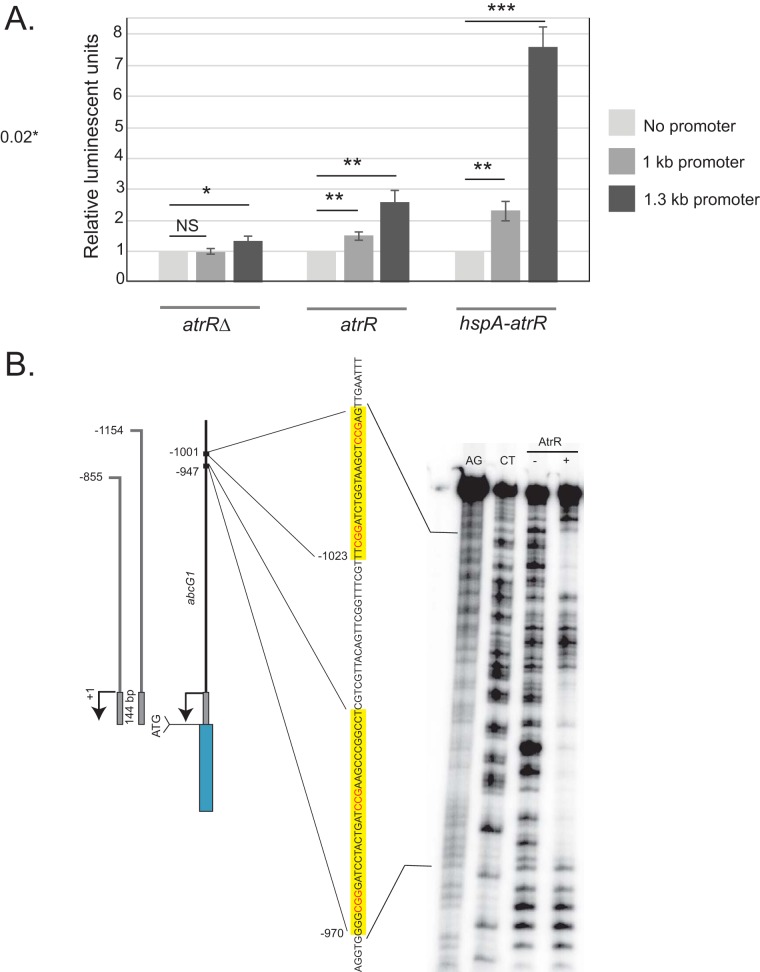
AtrR binding and function at *abcG1*. (A) Three different *abcG1*-firefly luciferase (fLUC) reporter genes were integrated into the following three indicated isogenic genetic backgrounds: *atrR*Δ mutant, wt (*atrR*), and an AtrR-overproducing strain (*hspA-atrR* mutant) at the *hisB* locus. Representative transformants were grown to mid-log phase, whole-cell protein extracts were prepared, and levels of firefly luciferase were assayed using a standard method as described previously (14). The promoter sequence present is denoted as no-promoter (only fLUC cassette), 1 kb or 1.3 kb of *abcG1* 5′ noncoding DNA (relative to the ATG). *P* values are indicated as described in the Fig. 2 legend. (B) DNase I protection analysis using recombinant AtrR and a cloned segment of the *abcG1* promoter (−1066 to −895 bp from the transcription start site) as a DNA probe. The protected regions are indicated on the sequence shown at the left in yellow boxes. The transcription start site is shown by the arrow, and the ATG and the start of the coding sequence for *abcG1* (blue box) are indicated.

Our finding of AtrR binding to a region more than 1,000 bp upstream of the ATG by ChIP-seq suggested a possible explanation for this observation, as we may have inadvertently truncated the *abcG1* promoter in our original construct. To test this hypothesis, we produced a new fusion construct that extended to 1,300 bp upstream of the ATG and contained the predicted ATREs for *abcG1*. We introduced this longer construct, along with our original fusion and a no-promoter control into wild-type, *atrR*Δ mutant, and *hspA-atrR* mutant cells. Representative transformants were grown overnight and then levels of *abcG1* promoter-driven luciferase assayed (Fig. 8A).

The 1.3-kb promoter region was expressed more highly than the 1-kb construct in all genetic backgrounds. This is most dramatically seen in the presence of the hypermorphic allele of *atrR* in which luciferase levels of the 1.3-kb reporter construct were nearly 4-fold elevated compared to those of the 1-kb fusion gene. The increase in luciferase levels when the 1-kb construct was assayed in the *hspA-atrR* background compared to the wild-type was less than 2-fold. This supports the notion that the region from 100 to 1,300 bp upstream of the ATG of *abcG1* may contain the AtrR-responsive region.

Sequence inspection of this same upstream region showed the presence of two potential ATREs (see Table 1). We carried out DNase I protection analysis of this portion of the *abcG1* promoter using recombinant AtrR as described above for *cyp51A* (Fig. 8B). This analysis demonstrated the presence of two ATREs in this region of the *abcG1* promoter and provides a molecular understanding for the defective AtrR response of the 1-kb reporter gene that lacks these two elements.

**TABLE 1:** *A. fumigatus* strains used in this study

Strain	Parent	Genotype	Reference or source
AfS35	D141	*akuA*Δ::*loxP*	FGSC
SPF87	AfS35	*atrR*Δ::*ptrA*	This study
SPF89	AfS35	*atrR-*3× HA::*hph*	13
SPF108	AfS35	*ptrA*::*hspA-atrR*	This study
SPF112	SPF89	*ptrA*::*hspA-atrR-*3× HA::*hph*	This study
SPF84	AfS35	*abcG1*Δ::*loxP*	16
SPF110	SPF84	*abcG1*Δ *ptrA*::*hspA-atrR*	This study
SPF134	AfS35	*srbA*Δ::*hph*	This study
SPF137	SPF108	*srbA*Δ *ptrA*::*hspA-atrR*	This study
SPF114	SPF87	*atrR*Δ *hisB*Δ::*fLUC*::*hph*	This study
SPF116	SPF87	*atrR*Δ *hisB*Δ::*1kb abcG1-fLUC*::*hph*	This study
SPF118	SPF87	*atrR*Δ *hisB*Δ::*1.3kb abcG1-fLUC*::*hph*	This study
SPF119	AfS35	*hisB*Δ::*fLUC*::*hph*	This study
SPF121	AfS35	*hisB*Δ::*1kb abcG1-fLUC*::*hph*	This study
SPF123	AfS35	*hisB*Δ::*1.3kb abcG1-fLUC*::*hph*	This study
SPF124	SPF108	*hspA-atrR hisB*Δ::*fLUC*::*hph*	This study
SPF126	SPF108	*hspA-atrR hisB*Δ::*1kb abcG1-fLUC*::*hph*	This study
SPF128	SPF108	*hspA-atrR hisB*Δ::*1.3kb abcG1-fLUC*::*hph*	This study

### The expression level of AtrR is important during pathogenesis.

**FIG 9:**
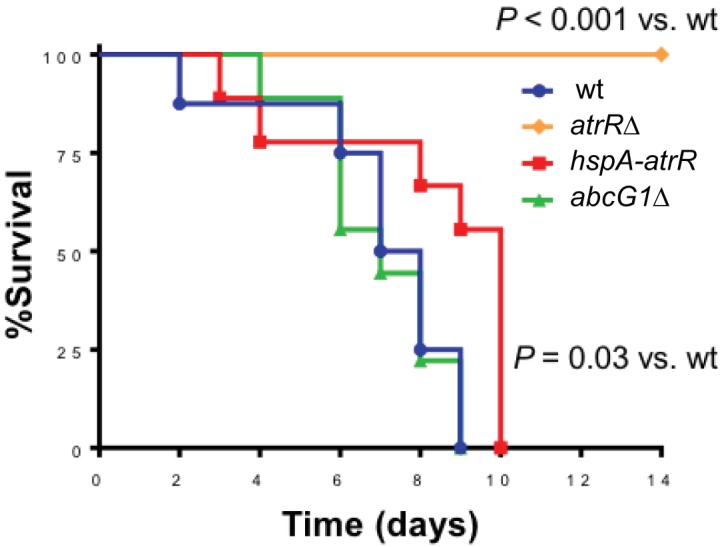
Effect of *atrR* alleles and *abcG1*Δ mutation on virulence. Eight mice per strain of *A. fumigatus* were immunosuppressed with cortisone acetate and then infected in an aerosol chamber and monitored for survival. Note that PBS control data are not distinguishable from *atrR*Δ infection. *P* for this experiment, <0.0001, *P* for pairwise comparison of *hspA-atrR* and wt = 0.03, by the log-rank rank sum test.

A loss of *atrR* was previously shown to strongly reduce virulence in a mouse model of aspergillosis (13). Here, we have described two new hyperactive alleles of *atrR* and wanted to determine what impact these would have on virulence in this murine model. We used a lung inhalation model (25) to test the impact of loss of a key AtrR target gene, the ABC transporter-encoding *abcG1* locus, or overproduction of AtrR itself (*hspA-atrR*) on fungal virulence. Mice were challenged with these strains along with an isogenic wild-type control and survival followed over an 11-day time course (Fig. 9).

We found that elevating the expression of AtrR via insertion of the *hspA* promoter led to a delay in mortality caused by this strain. A loss of *atrR* rendered the resulting mutant strain avirulent, as reported before. In this model, loss of the *abcG1* gene did not have an observable effect on virulence, contrary to results in found in a Galleria model of infection (16). These data argue that the precise level of AtrR is crucial for normal virulence. Normal pathogenesis thus requires a particular optimal dosage of AtrR with either elevated or depressed levels causing virulence defects in the animal model, a so-called “Goldilocks effect.”

## DISCUSSION

Our identification of the ATRE as a component of the 34-bp region in the *cyp51A* promoter adds an important new element to an already crowded regulatory region. As seen for SRE1 (9), the ATRE is duplicated in the TR_34_ repeat, consistent with this AtrR being a key contributor to the elevated gene transcription found in the presence of this promoter alteration. A second common *cyp51A* promoter variant, called TR_46_ (26), would also contain two SREs and two ATREs, supporting the view that the duplication of binding sites for both of these factors is required for the observed increment in gene expression in both TR mutants. Further analysis of the TR_34/46_ duplications is required to determine which parts of these duplicated promoters are hyperactive. At least in the case of the TR_34_ promoter, although the CBC recognition site is duplicated, it does not appear to be a functional binding site (9). This suggests that only the downstream CBC/HapX-repressive interaction is operational and possibly that the upstream duplicated 34-bp region is free of this normal repression.

The steady-state accumulation of AtrR at the protein level is sensitive to the form of this factor that is produced. The presence of the 3× HA epitope tag at the C terminus of AtrR caused the protein to behave as a hypermorphic allele and to exhibit lowered steady-state levels compared to the wild-type factor. Even when *atrR*-3× HA is driven by the *hspA* promoter, the resulting steady-state level is almost the same as when driven by the native *atrR* promoter. We cannot eliminate the possibility that replacement of the wild-type *atrR* promoter with that of the much stronger *hspA* element fails to increase the transcription of *atrR*-3× HA, although this is seen to occur in a comparison of wild-type *atrR* and *hspA-atrR*. Hyperactive transcription factors have been associated with more rapid degradation through ubiquitin-dependent means (27), and we speculate that this is true for the AtrR-3× HA protein. These data are consistent with the insertion of the 3× HA tag causing some alteration in the structure of AtrR that blocks proper control by a presumptive negative regulatory signal. We argue that since the expression level of AtrR is not increased at the protein or mRNA level, the elevated transcription rate that the AtrR-3× HA supports is due to a change in the activity of this protein. Similarly, elevated expression of the native factor appears to exceed the capacity of this negative regulation to restrain the transcriptional activation of AtrR. These suggestions are consistent with the hypothesis that the cause of hyperactivity of these two different *atrR* alleles is not the same. Both of these observations are consistent with the notion that AtrR is normally subject to negative regulation.

The large number of genes containing ChIP-seq peaks corresponding to AtrR was an unexpected observation. Earlier work identified approximately 120 genes as containing SREs and being transcriptionally responsive to SrbA (18, 22). Our ChIP-seq analysis detected nearly 900 genes with a significant association with AtrR, but fewer than 20% of these were induced in the presence of either hypermorphic allele of *atrR* used. Approximately 200 (*atrR*-3× HA) or 600 (*hspA-atrR*) genes were respectively induced in hypermorphic *atrR* backgrounds, but a minority of these induced genes contained ATREs. This is most readily interpreted by the majority of the transcriptional induction seen in these strains resulting from indirect effects. We hypothesize that AtrR occupies a relatively upstream position in the network of transcription factors that impact gene regulation in A. fumigatus, as supported by the GO term enrichment for these factors in the ChIP-seq data (Table S1). When AtrR is activated, it can induce the expression and function of a number of other transcriptional regulatory proteins and the circuits they directly control. We suggest that the full spectrum of transcriptional responses to changes in AtrR activity consist of a relatively smaller number of direct target genes (i.e., *abcG1* and *cyp51A*) and a larger collection of genes that are activated via AtrR control of genes encoding other transcription factors (i.e., *srbA* and *srbB*).

While our data strongly support the ability of a sequence element with the CGG(N)_12_CCG as being sufficient to function as an ATRE *in vivo*, this single consensus is not sufficient to explain the entire range of target genes bound by AtrR. As can be seen in Table 2 and Fig. 3B, the precise consensus for *in vivo* ATREs is likely to be more complex and will require further characterization. Our finding of the important tandem ATREs located more than 1 kb upstream of the ATG in *abcG1* illustrates the potentially increased complexity for A. fumigatus promoter organization compared to similar regions in more extensively characterized fungi, such as Saccharomyces cerevisiae (reviewed in reference 28).

**TABLE 2:** ATREs from selected genes^*a*^

Upstream endpoint	ATRE	Downstream endpoint	Locus
−308	**CGG**ATGTGTGCTGAG**CCG**	−291	*cyp51A*
−842	**CGG**GGTTAACGGCTT**CCG**	−825	*atrR*
−1132	**CGG**AGCTTACCAGAT**CCG**A	−1114	*abcG1*
−1183	**CGG**ATCAGTAGGATC**CCG**	−1166	
−754	**CGG**AATCGCTTCTTC**CCG**	−737	*srbA*
−617	**CGG**TAGCCATGATGAt**CG**	−600	*erg25*
−770	**CGG**AGTTTCGTCCAAt**CG**	−753	*erg5*
−1081	**CGG**CCATTCGGATAT**CCG**	−1064	*erg6*

a Numbers refer to distance upstream of the respective ATG, while the bolded letters indicate the conserved triplet.

Finally, the hypovirulent phenotype caused by either loss of *atrR* or overproduction of the wild-type factor illustrates the importance of the dosage of AtrR during pathogenesis. The normal disease process required a properly metered level of this protein and suggests that interventions targeting the activity of this transcriptional regulator might be effective in interfering with the pathogenicity of this important fungus. The essential role AtrR plays in azole resistance would also be impacted by interfering with normal function of this transcription factor. Recent experiments describing the successful identification of a drug preventing normal transcription factor activation (29) suggest that this strategy might be effective to reduce AtrR-dependent transactivation as well. Further understanding of the molecular details explaining AtrR control of gene expression is essential to develop strategies to accomplish this goal.

## MATERIALS AND METHODS

### Strains and growth conditions.

All strains used in this study were derived from the AfS35 (FGSC #A1159) strain and are listed in Table 1. A. fumigatus strains were typically grown at 37°C in rich medium (Sabouraud dextrose; 0.5% tryptone, 0.5% peptone, 2% dextrose [pH 5.6 ± 0.2]). Selection of transformants was made in minimal medium (MM; 1% glucose, nitrate salts, trace elements, 2% agar [pH 6.5]); trace elements, vitamins, and nitrate salts are as described in the appendix of reference 30, supplemented with 1% sorbitol and either 200 mg/liter hygromycin Gold (InvivoGen) or 0.1 mg/liter pyrithiamine. For solid medium, 1.5% agar was added. For both ChIP- and RNA-seq experiments, all cells were cultured on rich Sabouraud dextrose medium as biofilms prior to the isolation of either fixed chromatin or RNA, respectively.

Derivatives containing the *hspA-atrR* construct were made as follows: 4 DNA fragments consisting of 1,000 bp upstream of the *atrR* coding sequence, β-rec/six-*ptrA* blaster cassette (from pSK485), *hspA* promoter, and 1,000 bp corresponding to the *atrR* 5′ coding sequence were joined by Gibson assembly at the BamH1 site of pUC19 to form pSP99. This plasmid was then digested with SmaI and PmeI to release the 8.5-kb integration construct from the plasmid backbone and transformed into AfS35, SPF89, and SPF844 to generate strains SPF108, SPF112, and SPF110, respectively, under pyrithiamine selection.

The *srbA*Δ disruption strain was generated using a hygromycin split-marker strategy (31). The split-marker cassettes were constructed by fusion PCR. The upstream split marker had a 1.2-kb region corresponding to the DNA segment immediately upstream of the *srbA* gene, followed by a partial hygromycin cassette. The downstream split marker had a 1.2-kb DNA fragment corresponding to the region immediately downstream of the *srbA* gene preceded by the complementary partial hygromycin cassette. The hygromycin partial cassettes contained a 600-bp overlap between the split markers. The upstream and downstream split markers were cotransformed into the AfS35 and the SPF108 strain to generate SPF134 and SPF137, respectively.

The *abcG1* promoter-luciferase strains were generated as follows: an A. fumigatus *hisB* (Afu6g04700) targeting construct was initially made in a pRS316 vector backbone containing 1.2 kb DNA upstream of the *hisB* gene, the complete hygromycin cassette, firefly luciferase gene (from the pGL3 plasmid [Promega Corp., Madison, WI]), and 1.2-kb DNA downstream of the *hisB* gene using Gibson assembly. This plasmid was named pSP62 and was digested with PmeI and AscI to insert either the 1-kb or 1.3-kb *abcG1* promoter fragment just upstream of the luciferase reporter gene to generate pSP101a and pSP101b. The pSP62, pSP100a, and pSP100b plasmids were digested with NotI and KpnI to release the integration construct from the vector and transformed in SPF87, AfS35, and SPF108 strain backgrounds to generate the different transcriptional fusion reporter strains given in Table 1.

### Drug diffusion/spot assay.

Fresh spores of A. fumigatus were suspended in 1× phosphate-buffered saline (PBS) supplemented with 0.01% Tween 20 (1× PBST). The spore suspension was counted using a hemocytometer to determine the spore concentration. Spores were then appropriately diluted in 1× PBST. For the drug diffusion assay, ∼10^6^ spores were mixed with 10 ml soft agar (0.7%) and poured over 15 ml regular agar containing (1.5%) minimal medium. A paper disk was placed on the center of the plate, and 10 μl of either 1 mg/liter voriconazole or 20 mg/liter fluconazole was spotted onto the filter paper. For the spot assay, ∼100 spores (in 4 µl) were spotted on minimal medium with or without the drug. The plates were incubated at 37°C and inspected for growth every 12 h.

### Generation of an AtrR antibody.

A region of 597 bp (corresponding to the 199 N-terminal amino acids) from *atrR* were PCR amplified and cloned in frame as BamHI/SalI fragments downstream of the 6×-His tag in pET28a+ (EMD Millipore, Inc.) to form pGT3 and transformed into the bacterial strain Escherichia coli BL21(DE3). Two liters of transformed bacteria was grown to log phase and induced with isopropyl-β-d-thiogalactopyranoside (IPTG) for 90 min. Cell lysates were prepared using a French press. Protein purification was accomplished using Talon metal affinity resin (TaKaRa Bio USA, Inc.) as described by the manufacturer. Protein fractions were analyzed by staining them with Coomassie blue and by Western blotting using His-specific antibodies. The purified proteins were then lyophilized and sent to Pacific Immunology (Ramona, CA) for injection into rabbits to generate polyclonal antibodies against AtrR. Antiserum generated from these rabbits was received and tested for immunoreactivity against A. fumigatus cell lysates. The antiserum was then purified using an AminoLink Plus coupling resin (Thermo Scientific, Inc.) according to the manufacturer’s instructions, and the affinity-purified antiserum was used to detect the AtrR protein from A. fumigatus cell lysates.

### Western blotting.

Western blotting was performed as described in reference 32. The rabbit AtrR polyclonal antibody was used at a 1:100 dilution, while the anti-AbcG1 antibody was used at a 1:1,000 dilution.

### Real-time PCR.

Quantitative reverse transcription-PCR (qRT-PCR) was performed as described in reference 32, with the exception that cell lysates were prepared from mycelial biofilm cultures formed upon inoculating 10^6^ spores in a petri dish containing 20 ml of Sabouraud dextrose broth and grown for 24 h at 37°C under nonshaking conditions. The threshold cycle (*C_T_*) value of the *act1* transcript was used as a normalization control.

### ChIP-seq.

Chromatin immunoprecipitation (ChIP) was done as described in reference 13. Purified ChIP-ed DNA was then subjected to library preparation as outlined in reference 33, with the following modifications. The purified ChIP-ed DNA was concentrated to 10 μl using a SpeedVac system and the libraries set up using the Rubicon ThruPLEX DNA-seq kit with 10 μl as the input volume. The paired reads were mapped to the Af293 A. fumigatus genome using Bowtie 2 (34). The MACS2 algorithm was used to call significant read depth peaks upon comparing epitope-tagged samples to the untagged wild-type control (35). ChIP-seq peaks were analyzed for proximity to annotated protein-coding genes using the ChIPPeakAnno package (36). A fasta file was generated containing 250 bp on each side of significant peak summits and analyzed for conserved motifs using MEME-ChIP (37). A summary of all next-generation sequencing (NGS) data (ChIP- and RNA-seq) is provided in Table S2.

10.1128/mBio.02563-18.4 TABLE S2 ChIP- and RNA-seq data sets from strains producing various forms of AtrR. This table summarizes both NGS data sets generated in the production of the manuscript. Columns A to U correspond to the ChIP-seq data that led to our identification of the AtrR target genes, and columns W to Y are the corresponding RNA-seq expression data as log_2_ values. Expression values are listed as ratios of RNA levels as in the table legend above. Column A has the designated peak names for each peak called by MACS2; columns B and C are the peak start and end values along the chromosome; column D is the width of the peak region; column E is the position of the top value of read counts (abs_summit); column F is the −log_10_(false-discovery rate) for each peak; column G is the fold enrichment of read counts in the HA-tagged strain compared to the untagged wild-type control; column H lists the length of each identified peak; column I is the −log_10_(*q* value) corresponding to the fold enrichment for the peaks; columns J and K are the average and maximal −log_10_(*q* value) for the peaks identified in a given length, respectively; columns L and M represent the start and stop positions of the nearby feature (typically a gene), respectively; column N indicates the strand location of a feature; column O is the relative location of a peak compared to the feature of interest; column P is the distance of the most upstream peak to the feature, while column Q is the distance from the peak closest to the feature; columns R and S are the systematic or common gene names for each feature; column T is the description of the function of the encoded product, and column U indicates if this feature has been verified for expression. AC to AG represent the RNA-seq data for the strains described above. The systematic name is in AC and the length of the related transcript in is column AD. Download Table S2, XLSX file, 0.9 MB. Copyright © 2019 Paul et al. 2019 Paul et al. This content is distributed under the terms of the Creative Commons Attribution 4.0 International license.

### RNA-seq.

RNA was prepared as described in reference 32, with the exception that cell lysates for RNA were prepared as described above for generating samples for qRT-PCR. RNA samples were quantified using fluorimetry, and RNA quality was assessed using the Agilent 2100 Bioanalyzer. Sequencing libraries were generated using the TruSeq stranded mRNA sample preparation kit (Illumina, Inc., San Diego, CA) and, following Illumina’s sample preparation guide, started with 500 ng of input total RNA. The molar concentrations of the indexed libraries were measured using the 2100 Bioanalyzer (Agilent Technologies, Santa Clara, CA) with a high-sensitivity chip, and the libraries were combined equally into one pool. The molar concentration of the pool was measured using the Kapa Illumina library quantification kit (Kapa Biosystems, Wilmington, MA), and the pool was sequenced on one lane of the Illumina HiSeq 4000 sequencer with 75-bp paired-end SBS chemistry (Illumina). Paired-end reads were mapped to the Af293 A. fumigatus genome using HISAT2 (38). Transcript assembly, quantification of expression levels, and analysis of differential expression were done using StringTie and Ballgown (39).

### EMSA.

Radiolabeled EMSA was performed using the protocol described in reference 40, with the following modifications: a probe corresponding to the *cyp51A* promoter was PCR amplified using the GAAAAAACTCATGAGTGAATAATCG (sense [S]) and CATGCTGTATTTTATATTCACCTACC (antisense [AS]) primer pair. This region contained the 34 bp that is duplicated in the *cyp51A* TR_34_ allele and is associated with clinically relevant azole-resistant strains. The *atrR* probe was amplified using the CTAGATCCTGGATTCATGGCTC (S) and CGACTAACCACAATCGACTGG (AS) primer pair, the *abcG1* probe amplified using the TGTACGGAGTATATCCATAGCTG (S) and GTGACAAAAACTCCGCTAGCA (AS) primer pair, and the *srbA* probe amplified using the AGGGCAGGTAACGTTACAGCC (S) and GAATTGCTGCCTGTGCCTGAG (AS) primer pair. All of these probes were designed such that they corresponded to the AtrR ChIP-seq peak region for their respective promoters. Aliquots of each probe were end-labeled with [γ-^32^P]-ATP and T4 polynucleotide kinase. For competitive binding, 40-fold molar excess of the nonradiolabeled probe was added to the binding reaction.

Biotin-labeled EMSA was performed as follows: biotin-labeled oligonucleotides used in the reaction corresponded to the 34-bp sequence in the *cyp51A* promoter that is duplicated (TR_34_) in many drug-resistant clinical isolates and were ordered from IDT (Coralville, IA). The sense strands of the competitor nonlabeled oligonucleotides are listed in Fig. 7B. The complementary strands of the labeled and unlabeled oligonucleotides were annealed by heating at 95°C and gradual cooling to room temperature prior to use as probes. The binding reaction for recombinant AtrR-DNA binding domain (AtrR-DBD; 0.1 μg/reaction) was done in the presence of 25 mM Tris-HCl (pH 7.5), 80 mM NaCl, 35 mM KCl, 5 mM MgCl_2_, 10% glycerol, and 1 μg poly(dI-dC) in a 20-μl reaction volume. Twenty picomoles biotinylated probe was used per reaction. When nonlabeled probes were used, they were in 12-fold molar excess to the labeled probe. Incubation was done at room temperature for 30 min. DNA and protein-DNA complexes were resolved by electrophoresis at 100 V on a 5% native acrylamide gel at room temperature using a buffer containing 45 mM Tris (pH 8.0), 45 mM boric acid, and 1 mM EDTA (TBE). DNA was electrotransferred from the gel to a nylon membrane (Hybond-*N* + Amersham) in 0.5× TBE buffer at 100 V for 30 min. The membrane was then blocked in 1% SDS in Odyssey blocking buffer (PBS) for 30 min, replaced with fresh blocking buffer plus 1% SDS, and incubated in 1:10,000 streptavidin-680 nm IRDye (Li-Cor) for 30 min at room temperature. The membrane was then washed three times in 1× PBST for 5 min each, followed by scanning using a Li‐Cor Odyssey infrared imaging system, application software version 3.0, and quantified using the Image Studio Lite 4.0 software (Li‐Cor).

### DNase I footprinting.

DNase I footprinting assay was carried out as described in reference 40, with the following modifications: for generation of the *cyp51A* probe, a PCR product corresponding to 453 to 191 bp upstream of the translation start site containing the 34 bp duplicated in TR_34_ was cloned into pCR2.1-TOPO. This plasmid was digested with SalI, treated with calf intestinal alkaline phosphatase, phosphorylated with T4 polynucleotide kinase and [γ-^32^P]-ATP, and finally cleaved with HindIII. For generation of the *abcG1* probe, a PCR product of the *abcG1* promoter containing 1,210 to 1,039 bp upstream of the *abcG1* translation start site, including the ChIP-seq peak region, was cloned into pCR2.1-TOPO. This plasmid was digested with SalI, treated with calf intestinal alkaline phosphatase, phosphorylated with T4 polynucleotide kinase and [γ-^32^P]-ATP, and finally cleaved with MluI.

### Luciferase assay.

Firefly luciferase assays were done using the firefly luciferase assay kit procured from Biotium, Inc., as described in reference 14.

### Mouse model of invasive aspergillosis.

To investigate the virulence of various strains, we used a nonneutropenic mouse model of invasive aspergillosis (41). Briefly, 6-week-old and 20- to 22-g male BALB/c mice (Taconic Laboratories) were immunosuppressed with 7.5 mg cortisone acetate (Sigma-Aldrich) administered subcutaneously every other day from day 4 before infection to day 4 after infection, for a total of 5 doses. To prevent bacterial infection, enrofloxacin (Baytril; Western Medical Supply) was added to the drinking water at a final concentration of 0.005% the day before beginning the cortisone acetate treatment. Next, 11 to 12 mice per group were infected in an acrylic chamber exposing them for 1 h to an aerosol generated from 12 ml of PBS containing 10^9^ conidia/ml. Immediately after infection, 3 mice from each group were sacrificed, and lungs were harvested, homogenized, and quantitatively cultured to verify the delivery of conidia to the lungs. The remaining mice were monitored twice daily for survival assessment. As a negative control, 8 mice were immunosuppressed but not infected.

### Data availability.

All NGS data are available as a linked GEO SuperSeries accession number GSE123446.
