# Supplementary material for: AtrR Is an Essential Determinant of Azole Resistance in Aspergillus fumigatus
Source: mBio. 2019 Mar 12;10(2):e02563-18. doi: 10.1128/mBio.02563-18 (PMC6414702; doi:10.1128/mBio.02563-18)

All (14) significantly enriched categories

Method: GO

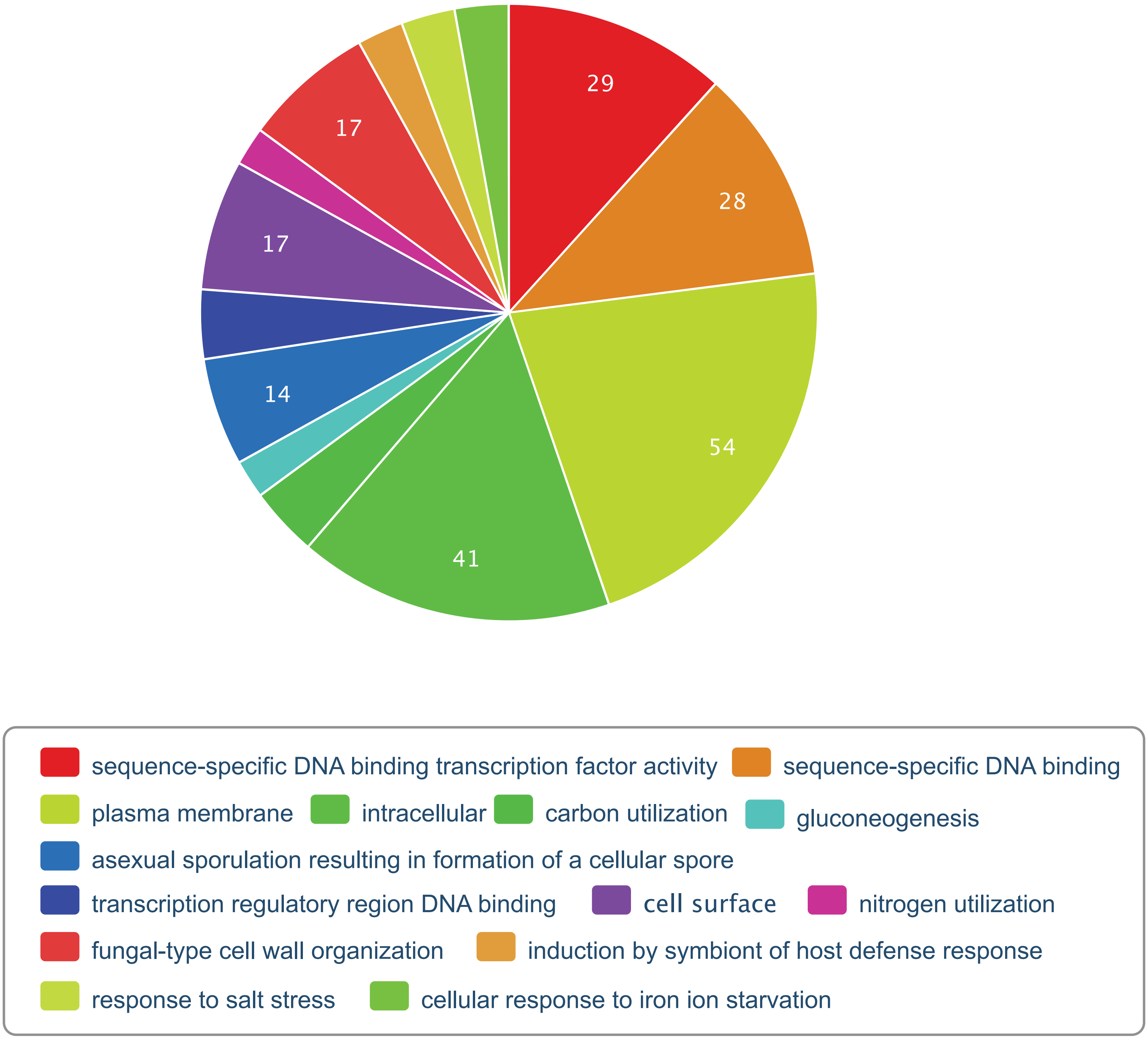

Supplement: FIG S1 [file mBio.02563-18-sf001.pdf]
