# Supplementary material for: AtrR Is an Essential Determinant of Azole Resistance in Aspergillus fumigatus
Source: mBio. 2019 Mar 12;10(2):e02563-18. doi: 10.1128/mBio.02563-18 (PMC6414702; doi:10.1128/mBio.02563-18)
Supplement: FIG S2 [file mBio.02563-18-sf002.pdf]

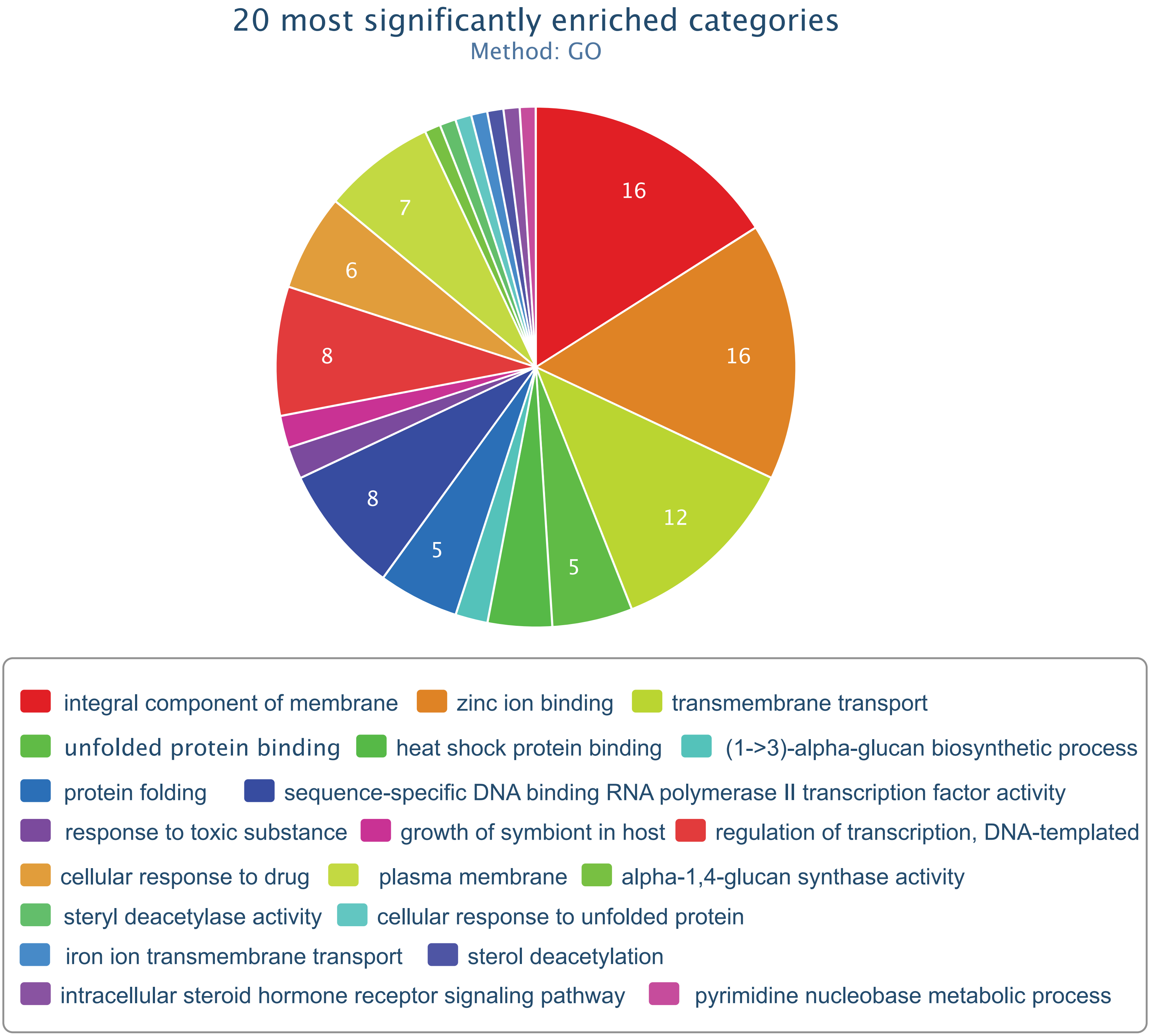
20 most significantly enriched categories
Method: GO
16
16
12
5
5
8
8
6
7
integral component of membrane
zinc ion binding
transmembrane transport
unfolded protein binding
heat shock protein binding
(1->3)-alpha-glucan biosynthetic process
protein folding
sequence-specific DNA binding RNA polymerase II transcription factor activity
response to toxic substance
growth of symbiont in host
regulation of transcription, DNA-templated
cellular response to drug
plasma membrane
alpha-1,4-glucan synthase activity
steryl deacetylase activity
cellular response to unfolded protein
iron ion transmembrane transport
sterol deacetylation
intracellular steroid hormone receptor signaling pathway
pyrimidine nucleobase metabolic process
